# ﻿Two new *Botryosphaeria* (Botryosphaeriales, Botryosphaeriaceae) species in China

**DOI:** 10.3897/mycokeys.94.91340

**Published:** 2022-11-11

**Authors:** Jing-E Sun, Chao-Rong Meng, Alan J. L. Phillips, Yong Wang

**Affiliations:** 1 Department of Plant Pathology, Agricultural College, Guizhou University, Guiyang, 550025, China Guizhou University Guiyang China; 2 Faculty of Sciences, Biosystems and Integrative Sciences Institute (BioISI), University of Lisbon, Campo Grande, 1749-016 Lisbon, Portugal University of Lisbon Campo Grande Portugal

**Keywords:** Ascomycetes, molecular analyses, morphology, new species, new woody host

## Abstract

Five ascomycetous strains were isolated from dead branches and leaves of *Salix* (Salicaceae) and *Osmanthusfragrans* (Oleaceae), respectively. BLAST searches with ITS sequences in GenBank suggested a high degree of similarity to *Botryosphaeriadothidea*. To accurately identify these strains, we further analysed their morphological characteristics of asci, ascospores, all conidiophore cells and conidia. Phylogenetic relationships, based on ITS, *rpb2*, *tef1* and *tub2* gene sequences, confirmed our strains represented two novel species, which are introduced here as *B.salicicola* and *B.osmanthuse***spp. nov.**

## ﻿Introduction

The genus *Botryosphaeria* (Botryosphaeriales, Botryosphaeriaceae) was established by Cesati and Notaris (1863) and is widely distributed throughout many geographical and climatic regions of the world, with the exception of polar regions (Phillips et al. 2013). Species of *Botryosphaeria* are reported in many woody plants as endophytes, saprobes and pathogens (Crous et al. 2006; Liu et al. 2012; Phillips et al. 2013; Ariyawansa et al. 2016; Dissanayake et al. 2016; Slippers et al. 2017). Some species of *Botryosphaeria* are aggressive pathogens that pose a significant threat to agricultural and forest ecosystems (Slippers and Wingfield 2007). *Botryosphaeriadothidea* is known to cause serious diseases, such as Apple ring rot (Slippers and Wingfield 2007; Marsberg et al. 2017). Moreover, according to the database of the common names of plant diseases in Japan, 14 species of the genus *Botryosphaeria* cause diseases on 30 plant species (Yukako et al. 2021).

*Botryosphaeria* has been considered as one of the hot topics in fungal taxonomy for a long time, based on its universality, including areas and hosts (from 1863 to 2022) (Cesati and Notaris 1863; Shoemaker 1964; Pennycook and Samuels 1985; Slippers et al. 2004; Slippers and Wingfield 2007; Liu et al. 2012; Phillips et al. 2008, 2019; Xu et al. 2015; Ariyawansa et al. 2016; Zhou et al. 2016, 2017; Li et al. 2018, 2020; Vu et al. 2019; Chen et al. 2020; Chu et al. 2021; Yukako et al. 2021). More than 300 species epithets are listed in MycoBank (https://www.mycobank.org, 17 October 2022), but only about 7% of *Botryosphaeria* species currently have associated DNA sequences data. In the past, species in *Botryosphaeria* were defined, based on morphological characters alone or on host association, but studies have shown these are inadequate characters to identify species (Shoemaker 1964; Pennycook and Samuels 1985; Slippers et al. 2004). With the advent of DNA sequencing methods, the nomenclature and identification of *Botryosphaeria* species have significantly improved (Phillips et al. 2013).

Some species of *Botryosphaeria* are aggressive pathogens in China, mainly distributed in the southwest, such as *B.fabicerciana*, *B.fujianensis*, *B.fusispora*, *B.kuwatsukai*, *B.dolichospermatii*, *B.pseudoramosa* and *B.wangensis* as shown in Table 4. In this study, five strains were isolated during surveys of fungi on new woody hosts (Salicaceae and Oleaceae) in Guizhou and Guangxi Provinces, China. Combining morphology and phylogenetic analyses, these isolates represent two novel *Botryosphaeria* species, which are described and illustrated here. The discovery of new species within this genus is important to help researchers better understand the diversity and ecology of *Botryosphaeria*.

## ﻿Materials and methods

### ﻿Sampling, fungal isolation and morphological observation

Fungi were isolated from dry branches of *Salix* (Salicaceae) and diseased leaf pieces of *Osmanthusfragrans* (Oleaceae) collected in forest parks in Guizhou and Guangxi Provinces, China, respectively. Samples were placed in envelopes and returned to the laboratory as described by Senanayake et al. (2020). Fruiting bodies (including asci, ascospores, conidiophore cells and conidia) on natural substrates were observed using a Zeiss Scope 5 compound microscope Axioscope 5 (Carl Zeiss Microscopy GmbH, Jena, Germany) with the microscope techniques of differential interference contrast light (DIC) and photographed using an AxioCam 208 colour (Carl Zeiss Microscopy GmbH, Jena, Germany) camera and saved as JPG files. Approximately 30 measurements of new species were made of each feature using the ZEN 3.0 (blue edition) (Jena, Germany) software.

Pure cultures were obtained using a single spore isolation method as described in Senanayake et al. (2020). The germinated spores were transferred to fresh potato dextrose agar (PDA) plates and incubated at 25 °C for 14 days. Type specimens were deposited in the Herbarium of the Department of Plant Pathology, Agricultural College, Guizhou University (**HGUP**). Ex-type cultures were deposited in the Culture Collection at the Department of Plant Pathology, Agriculture College, Guizhou University, P.R. China (**GUCC**). Taxonomic information of the new species was submitted to MycoBank (www.mycobank.org).

### ﻿DNA extraction, PCR and sequencing

Mycelium growing on PDA for seven days was scraped off with a sterile scalpel. Total DNA was extracted with a (Biomiga#GD2416, San Diego, California, USA) BIOMIGA Fungus Genomic DNA Extraction Kit (GD2416) following the manufacturer’s protocol. Four loci (ITS, *rpb2*, *tef1* and *tub2*) were amplified with the respective forward and reverse primers (Table 1). PCR cycling conditions were followed according to Yukako et al. (2021). For ITS: initial denaturation (94 °C, 5 min), 40 cycles of amplification (denaturation 94 °C, 45 s; annealing 48 °C, 30 s; and extension 72 °C, 90 s) and final extension (72 °C, 2 min); for *tef1*: initial denaturation (94 °C, 5 min), 40 cycles of amplification (denaturation 94 °C, 30 s; annealing 52 °C, 30 s; and extension 72 °C, 45 s) and final extension (72 °C, 2 min); for *tub2*: initial denaturation (94 °C, 5 min), 40 cycles of amplification (denaturation 94 °C, 30 s; annealing 52 °C, 30 s; and extension 72 °C, 60 s) and final extension (72 °C, 2 min); and for *rpb2*: initial denaturation (95 °C, 5 min), touch-down amplification (5 cycles of 95 °C for 45 s, 60 °C for 45 s and 72 °C for 120 s; 5 cycles of 95 °C for 45 s, 58 °C for 45 s and 72 °C for 120 s; and 30 cycles of 95 °C for 45 s, 54 °C for 45 s and 72 °C for 120 s) and final elongation at 72 °C for 8 min. PCR products were sequenced by SinoGegoMax (Beijing, China).

**Table 1.** Primers used in this study.

Used genes	Primer	Direction	Sequence (5’–3’)	Reference
*tef1*	EF1-688	Forward	CGGTCACTTGATCTACAAGTGC	Alves et al. (2008)
	EF1-1251	Reverse	CCTCGAACTCACCAGTACCG	
ITS	ITS1	Forward	TCCGTAGGTGAACCTGCGG	White et al. (1990)
	ITS4	Reverse	TCCTCCGCTTATTGATATGC	
*tub2*	BT-2a	Forward	GGTAACCAAATCGGTGCTGCTTTC	Glass and Donaldson (1995)
	BT-2b	Reverse	ACCCTCAGTGTAGTGACCCTTGGC	
*rpb2*	fRPB2-5f2	Forward	GATGATAGAGATCATTTTGG	Liu et al. (1999)
	fRPB2-7cR	Reverse	CCCATAGCTTGTTTACCCAT	

### ﻿Phylogenetic analyses

Newly-generated sequences were deposited in GenBank. All the taxa used in the phylogenetic analyses are provided in Table 2. These sequences were compared with the GenBank database using the Basic Local Alignment Search Tool (BLAST) and available sequences of species in the genus containing ex-type or representative isolates were downloaded from GenBank and previous publications (Li et al. 2018, 2020; Vu et al. 2019; Chen et al. 2020; Chu et al. 2021; Yukako et al. 2021). Alignments for the individual locus matrices were generated with the online version of MAFFT v. 7.307 (Katoh et al. 2019). Ambiguous sequences at the start and the end were deleted and the alignments edited with MEGA6 (Tamura et al. 2013) for maximum alignment and minimum gaps. Sequence matrix v. 1.7.8 was used to concatenate the aligned sequences (Vaidya et al. 2011). *Neoscytalidiumdimidiatum* (CBS 145.78 and CBS 251.49) and *Cophinformaatrovirens* (MFLUCC 11-0425 and MFLUCC 11-0655) were used as outgroup. Maximum Likelihood (ML), Maximum Parsimony (MP) and Bayesian Inference (BI) were used to place the newly-discovered strains into a phylogenetic framework and estimate phylogenetic relationships with other *Botryosphaeria* spp.

**Table 2.** Taxa used for molecular phylogenetic analyses and their GenBank accession numbers. (T) = ex-type strains.

Species	Strain	Host	Country	GenBank accession numbers			
				ITS	*tef1*	*tub2*	*rpb2*
*Botryosphaeriaagaves*	CBS 133992^T^	*Agave* sp.	Thailand	JX646791	JX646856	JX646841	N/A
*B.agaves*	MFLUCC 10-0051	*Agave* sp.	Thailand	JX646790	JX646855	JX646840	N/A
*B.auasmontanum*	CMW 25413^T^	*Pinus* sp.	Namibia	KF766167	N/A	N/A	N/A
*B.corticis*	CBS 119047^T^	*Vacciniumcorymbosum*	USA	DQ299245	EU017539	EU673107	N/A
*B.corticis*	ATCC 22927	*Vaccinium* sp.	USA	DQ299247	EU673291	EU673108	N/A
*B.dothidea*	CBS 115476^T^	*Prunus* sp.	Switzerland	AY236949	AY236898	AY236927	N/A
*B.dothidea*	CBS 110302	*Vitisvinifera*	Portugal	AY259092	AY573218	EU673106	N/A
*B.fabicerciana*	CBS 127193^T^	*Eucalyptus* sp.	China	HQ332197	HQ332213	KF779068	N/A
*B.fabicerciana*	CMW 27121	*Eucalyptus* sp.	China	HQ332198	HQ332214	KF779069	N/A
*B.fujianensis*	CGMCC 3.19099^T^	*Vacciniumuliginosum*	China	MH491973	MH491977	MH562330	N/A
*B.fujianensis*	BJFUCC 180226-3	*Vacciniumuliginosum*	China	MW251380	MW251388	MW251379	N/A
*B.fusispora*	MFLUCC 10-0098^T^	*Entada* sp.	Thailand	JX646789	JX646854	JX646839	N/A
*B.fusispora*	MFLUCC 11-0507	*Caryota* sp.	Thailand	JX646788	JX646853	JX646838	N/A
*B.guttulata*	CGMCC3.20094^T^	N/A	China	MT327839	MT331606	N/A	N/A
*B.guttulata*	GZCC 19-0188	N/A	China	MT327833	MT331601	N/A	N/A
*B.kuwatsukai*	CBS 135219^T^	*Malusdomestica*	China	KJ433388	KJ433410	N/A	N/A
*B.kuwatsukai*	LSP 5	*Pyrus* sp.	China	KJ433395	KJ433417	N/A	N/A
*B.dolichospermatii*	CGMCC 3.19096^T^	*Vacciniumuliginosum*	China	MH491970	MH491974	MH562327	N/A
*B.dolichospermatii*	CGMCC 3.19097	*Vacciniumuliginosum*	China	MH491971	MH491975	MH562328	N/A
*B.minutispermatia*	GZCC 16-0013^T^	Dead wood	China	KX447675	KX447678	N/A	N/A
*B.minutispermatia*	GZCC 16-0014	Dead wood	China	KX447676	KX447679	N/A	N/A
***B.osmanthuse***	**GUCC 21433^T^**	**GUCC 21433**	**China**	**OL854215**	**OP650906**	**OP669376**	**OP650903**
***B.osmanthuse***	**GUCC 21433.1**	***Osmanthusfragrans***	**China**	**OL854216**	**OP650907**	**OP669377**	**OP650904**
***B.osmanthuse***	**GUCC 21433.2**	***Osmanthusfragrans***	**China**	**OL854217**	**OP650908**	**OP669378**	**OP650905**
*B.pseudoramosa*	CERC 2001^T^	*Eucalyptushybrid*	China	KX277989	KX278094	KX278198	MF410140
*B.pseudoramosa*	CERC 2983	*Melastomasanguineum*	China	KX277992	KX278097	KX278201	MF410143
*B.puerensis*	CSF6052 T	*Eucalyptusurophylla*	China	MT028569	MT028735	MT028901	MT029057
*B.qingyuanensis*	CERC 2946^T^	*Eucalyptushybrid*	China	KX278000	KX278105	KX278209	MF410151
*B.qingyuanensis*	CERC 2947	*Eucalyptushybrid*	China	KX278001	KX278106	KX278210	MF410152
*B.quercus*	MFLUCC:14-0459 T	*Quercus* sp.	Italy	KU848199	N/A	N/A	N/A
*B.ramosa*	CBS 122069^T^	*Eucalyptuscamaldulensis*	Bell Australia	EU144055	EU144070	KF766132	N/A
*B.ramosa*	CGMCC 3.18004	*Acacia* sp.	China	KX197073	KX197093	KX197100	N/A
*B.rosaceae*	CGMCC 3.18007^T^	*Malus* sp.	China	KX197074	KX197094	KX197101	N/A
*B.rosaceae*	CGMCC 3.18008	*Amygdalus* sp.	China	KX197075	KX197095	KX197102	N/A
***B.salicicola***	**GUCC 21230^T^**	***Salix***	**China**	**OL854218**	**OP669379**	**OP750032**	**N/A**
***B.salicicola***	**GUCC 21230.1**	***Salix***	**China**	**OL854219**	**OP669380**	**OP750033**	**N/A**
*B.scharifii*	CBS 124703^T^	*Mangiferaindica*	Iran	JQ772020	JQ772057	N/A	N/A
*B.sinensia*	CGMCC 3.17722^T^	*Populus* sp.	China	KT343255	N/A	N/A	N/A
*B.tenuispora*	MUCC 2900	*Aucubajaponica*	Japan	LC585276	LC585148	LC585172	N/A
*B.tenuispora*	MUCC 237^T^	*Leucothoefontanesiana*	Japan	LC585278	LC585150	LC585174	LC585196
*B.wangensis*	CERC 2298^T^	*Cunninghaminadeodara*	China	KX278002	KX278107	KX278211	MF410153
*B.wangensis*	CERC 2299	*Cunninghaminadeodara*	China	KX278003	KX278108	KX278212	MF410154
*Cophinformaatrovirens*	MFLUCC 11-0425 T	*Eucalyptus* sp.	Thailand	JX646800	JX646865	JX646848	N/A
*C.atrovirens*	MFLUCC 11-0655	*Eucalyptus* sp.	Thailand	JX646801	JX646866	JX646849	N/A
*Neoscytalidiumdimidiatum*	CBS 145.78^T^	*Homosapiens*	United Kingdom	KF531816	KF531795	KF531796	N/A
*N.dimidiatum*	CBS 251.49	*Juglansregia*	USA	KF531819	KF531797	KF531799	N/A

Note: Newly generated sequences are indicated in bold.

ML analysis was performed using IQ-TREE (Nguyen et al. 2015; Trifinopoulos et al. 2016) on the IQ-TREE web server (http://iqtree.cibiv.univie.ac.at, 17 October 2022). The MP analysis was implemented to test the discrepancy of the ITS, *rpb2*, *tef1* and *tub2* sequence datasets with PAUP v. 4.0b10 (Swofford 2002). Gaps were treated as missing data, which were interpreted as uncertainty of multistate taxa. Phylogenetic trees were generated using the heuristic search option with tree bisection re-connection (TBR) branch swapping. “Maxtrees” was set to 5000, the tree length (TL), consistency index (CI), homoplasy index (HI), retention index (RI) and rescaled consistency index (RC) were calculated. Bayesian Inference analysis was made with MrBayes 3.2.6 (Ronquist et al. 2012) based on a best substitution model for ITS: GTR+G, *rpb2*: K2P+I, *tef1*: HKY+G and *tub2*: HKY+G. BI was performed using six Markov Chain Monte Carlo runs for 5,000,000 generations, sampling every 1000 generations. The first 25% resulting trees were discarded as burn-in phase of each analysis.

MP, ML bootstrap support values greater than 70% and BI posterior probability values greater than 0.90 were denoted at the nodes and separated by “/”. Bootstrap values less than 70% and BI posterior probability values less than 0.90 were labelled with “_”.

## ﻿Results

**Figure 1.**
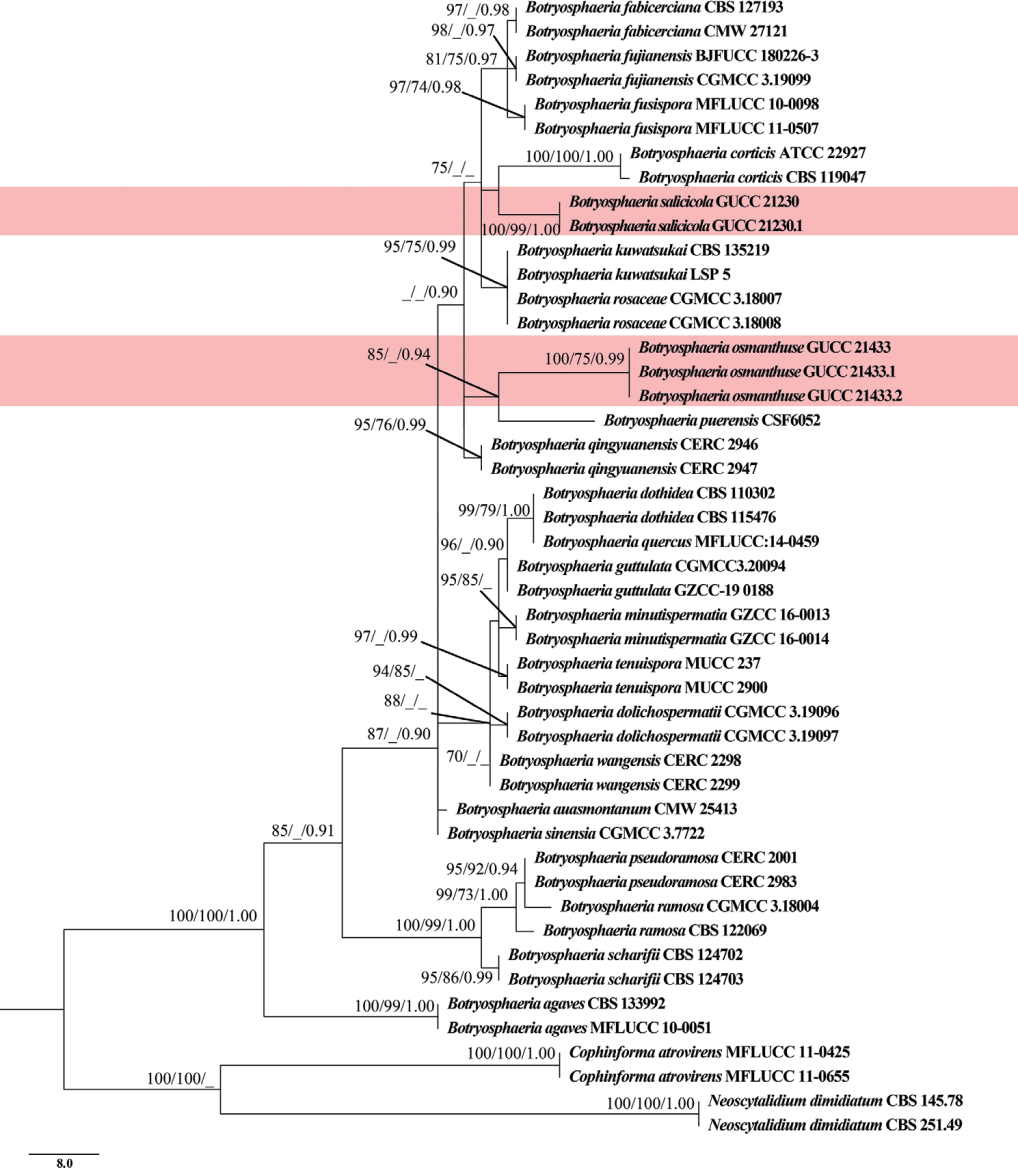
Trees resulting from MP analysis of the combined ITS, *rpb2*, *tef1* and *tub2* sequence alignment for forty-three isolates in *Botryosphaeria*. RAxML and MP bootstrap support values (ML, MP ≥ 70%) and Bayesian posterior probability (PP ≥ 0.90) are denoted on the nodes (ML/MP/PP). The tree was rooted to *Neoscytalidiumdimidiatum* (CBS 145.78 and CBS 251.49) and *Cophinformaatrovirens* (MFLUCC 11-0425 and MFLUCC 11-0655). The new species are highlighted in pale red. The scale bar indicates 8.0 expected changes per site.

The MP, ML and Bayesian analyses resulted in trees with similar topologies and the MP tree is shown in Fig. 1. The combined data matrix of ITS–*rpb2*–*tef1*–*tub2* consisted of 1805 characters (ITS: 466, *rpb2*: 716, *tef1*: 286 and *tub2*: 337), of which 1579 characters were constant and 13 variable characters were parsimony uninformative. Maximum Parsimony analysis of the remaining 213 parsimony informative characters produced a tree with the following parameters: TL = 291; CI = 0.862; HI = 0.137; RI = 0.931; and RC = 0.803.

In the phylogenetic tree (Fig. 1), the isolates from this study formed two distinct, well-supported clades and, thus, were considered to represent two previously unknown species. *Botryosphaeriaosmanthuse*GUCC 21433, GUCC 21433.1 and GUCC 21433.2 without the DNA base differences in four loci amongst strains (ITS, *rpb2*, *tef1* and *tub2*) form an independent branch with strong support (ML = 85, PP = 0.94) sister to *B.puerensis*. *Botryosphaeriasalicicola* (GUCC 21230 and GUCC 21230.1) clustered sister to *B.corticis*, *B.fabicerciana*, *B.fusispora*, *B.fujianensis*, *B.kuwatsukai* and *B.rosaceae*, although with weak-supports (ML = 75). These two novel taxa were also supported by DNA base pair differences (Table 3).

**Table 3.** The DNA base differences in four loci between the two new species and closely-related species.

Species	Strain number	ITS (1–458 characters)	*tef1* (459–703 characters)	*tub2* (704–1039 characters)	*rpb2* (1040–1754 characters)
*Botryosphaeriasalicicola*	GUCC 21230	0	0	0	–
	GUCC 21230.1	0	0	0	–
*B.corticis*	CBS 119047	10 (gap: 2)	11 (gap: 6)	6 (gap: 0)	–
	ATCC 22927	10 (gap: 2)	11 (gap: 6)	6 (gap: 0)	–
*B.fabicerciana*	CBS 127193	4 (gap: 3)	8 (gap: 2)	3 (gap: 1)	–
	CMW 27121	4 (gap: 3)	8 (gap: 2)	3 (gap: 1)	–
*B.fujianensis*	CGMCC 3.19099	4 (gap: 3)	8 (gap: 2)	4 (gap: 1)	–
	BJFUCC 180226-3	4 (gap: 3)	8 (gap: 2)	4 (gap: 1)	–
*B.fusispora*	MFLUCC 10-0098	4 (gap: 3)	10 (gap: 3)	3 (gap: 1)	–
	MFLUCC 11-0507	4 (gap: 3)	10 (gap: 3)	3 (gap: 1)	–
*B.kuwatsukai*	CBS 135219	4 (gap: 4)	7 (gap: 2)	–	–
	LSP 5	4 (gap: 4)	7 (gap: 2)	–	–
*B.rosaceae*	CGMCC 3.18007	4 (gap: 4)	7 (gap: 2)	2 (gap: 0)	–
	CGMCC 3.18008	4 (gap: 4)	7 (gap: 2)	2 (gap: 0)	–
*B.dothidea*	CBS 115476	8 (gap: 2)	12 (gap: 4)	3 (gap: 1)	–
	CBS 110302	8 (gap: 2)	12 (gap: 4)	3 (gap: 1)	–
**Species**	**Strain number**	**ITS (1–456 characters)**	***tef1* (471–702 characters)**	***tub2* (703–1034 characters)**	***rpb2* (1035–1750 characters)**
*Botryosphaeriaosmanthuse*	GUCC 21443	0	0	0	0
	GUCC 21443.1	0	0	0	0
	GUCC 21443.2	0	0	0	0
*B.puerensis*	CSF6052	1 (gap: 1)	13 (gap: 4)	8 (gap: 0)	8 (gap: 0)
*B.dothidea*	CBS 115476	5 (gap: 1)	9 (gap: 2)	12 (gap: 0)	–
	CBS 110302	5 (gap: 1)	9 (gap: 2)	12 (gap: 0)	–

### ﻿Taxonomy

#### ﻿ Botryosphaeria salicicola

Taxon classificationFungiBotryosphaerialesBotryosphaeriaceae

J. E. Sun, C. R. Meng & Yong Wang bis sp. nov.

BE2685D5-23B8-5A22-83E4-33062E56D79C

43685

Figs 2a–i

##### Etymology.

In reference to the host from which the fungus was first isolated.

##### Diagnosis.

*Botryosphaeriasalicicola* is characterised by oval to broadly fusiform ascospores (25.2 × 10.8; L/W = 2.3 vs. 22.7× 7.8 µm, L/W = 2.9) and cylindrical to clavate asci (65–170 × 20–30 µm), with moderate growth rate.

##### Type.

China, Guizhou Province, Guiyang City, 26°65'N, 106°63'E, from branches of *Salix* sp., 20 June 2020, C.R. Meng, HGUP 21230 (holotype), ex-type culture GUCC 21230.

##### Description.

Saprobic on dead branches of *Salix*. **Teleomorph: *Ascomata*** superficial, becoming erumpent at maturity, aggregated, thick-walled, wall composed of dark brown, thick-walled ***textura angularis***, becoming thinner-walled and hyaline towards the inner layers, 160 µm diam. ***Hamathecium*** comprising hyaline, septate, branched, 2–3.5 µm wide filamentous pseudoparaphyses. ***Asci*** 65–170 × 20–30 µm, 8-spored, bitunicate, cylindrical, to clavate, stipitate. ***Ascospores*** 22–26 × 9.0–13 µm (average = 25.2 × 10.8 µm, n = 20, L/W = 2.3), irregularly biseriate in the ascus, hyaline, guttulate, smooth with granular contents, aseptate, oval to broadly fusiform, widest in the middle or upper third of the ascospore, tapering to the obtuse base and apex. **Anamorph**: Not observed.

##### Culture characteristics.

Ascospores germinate on PDA within 24 hours at room temperature (25 °C). Colonies with white fluffy mycelium on PDA (90 mm), after 7 days becomes grey-black at the bottom of centre, olivaceous-grey at the bottom of edge, white mycelium, raised, fluffy, dense filamentous.

##### Distribution.

China, Guizhou Province, Guiyang City.

##### Other material examined.

China, Guizhou Provice, Guiyang City, 26°65'N, 106°63'E, from dead branches of *Salix*, 20 June 2020, C.R. Meng, HGUP 21230, living culture GUCC 21230.1.

##### Notes.

NCBI BLAST searches of ITS sequences from our strains suggested a high degree of similarity (99–100%) to *Botryosphaeriadothidea*. However, *B.salicicola* and *B.dothidea* show distant phylogenetic relationships in the phylogeny. *Botryosphaeriasalicicola* has longer asci (65–170 × 20–30 µm vs. 63–125 × 16–20 µm) than *B.dothidea* and longer ascospores (25.2 × 10.8; L/W = 2.3 vs. 22.7× 7.8 µm, L/W = 2.9) (Slippers et al. 2004). The phylogenetic analyses indicate that *Botryosphaeriasalicicola* forms an independent branch with respect to *B.corticis*, *B.fabicerciana*, *B.fusispora*, *B.fujianensis*, *B.kuwatsukai* and *B.rosaceae*. Comparing the morphological characteristics shows that *B.corticis* has longer ascospores than *B.salicicola* (29.3 × 11.6 µm vs. 25.2 × 10.8 μm) (Phillips et al. 2006); *B.fusispora* has shorter asci than *B.salicicola* (77.5–112.5 × 20–25 µm vs. 65–170 × 20–30 µm) (Liu et al. 2012); *B.rosaceae* has longer ascomata than *B.salicicola* (170–290 μm vs. 160 μm) (Zhou et al. 2017). The sexual morphs of *B.fabicerciana* (Chen et al. 2011), *B.fujianensis* (Chu et al. 2021) and *B.kuwatsukai* (Xu et al. 2015) are unknown.

**Figure 2.**
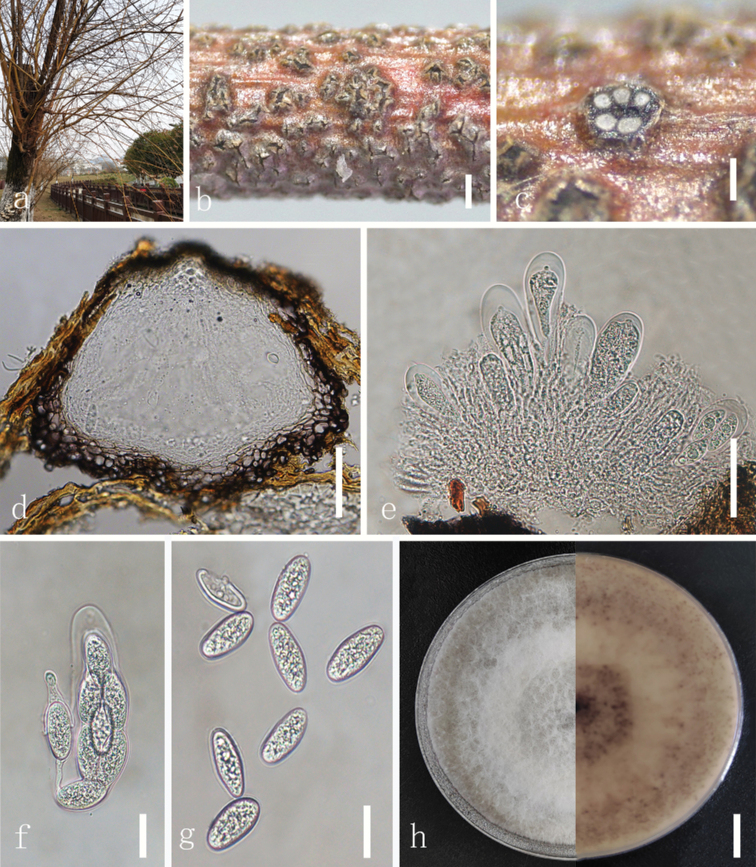
*Botryosphaeriasalicicola* (GUCC 21230, holotype) **a–c** ascomata on natural substrate **d** section through ascomata **f** mature asci **g** ascospores **h** colony on PDA (left: above, right: reverse). Scale bars: 400 µm (**b**); 200 µm (**c**); 50 µm (**d**); 40 µm (**e**); 20 µm (**f, g**); 15 mm (**h**).

#### ﻿ Botryosphaeria osmanthuse

Taxon classificationFungiBotryosphaerialesBotryosphaeriaceae

J. E. Sun, C. R. Meng & Yong Wang bis sp. nov.

943B4673-3B76-5616-A304-95694C73DF3F

843684

Figs 3a–i

##### Etymology.

In reference to the host from which the fungus was first isolated.

##### Diagnosis.

*Botryosphaeriaosmanthuse* is characterised by aseptate narrowly fusiform conidia (16.0–20.5 × 5.0–6.0 µm (average = 17.0 × 5.3 µm, n = 45, L/W = 3.2) and short-length conidiogenous cells (8.5–10.5 × 2.3–2.8 µm), with moderate growth rate.

##### Type.

China, Guangxi Province, Nanning City, 22°51'N, 108°19'E, from leaves of *Osmanthusfragrans*, 20 October 2017, C.R. Meng, HGUP 21433 (holotype), ex-type living culture GUCC 21433.

##### Description.

Saprobic on living leaves of *Osmanthusfragrans*. **Teleomorph**: Not observed. **Anamorph: *Conidiomata*** up to 200 µm diam., covered with hyphae, black, globose, ostiolate, solitary, separate, uniloculate, immersed to semi-immersed. ***Conidiomatal wall*** composed of thick-walled, dark brown cells of ***textura angularis***, becoming thin-walled and hyaline towards the inner region. ***Conidiophores*** reduced to conidiogenous cells. ***Conidiogenous cells*** 8.5–10.5 × 2.3–2.8 µm (average = 10 × 2.5 µm, n = 20), holoblastic, discrete, hyaline, cylindrical to lageniform, phialidic with periclinal thickening. ***Paraphyses*** not were seen. ***Conidia*** 16.0–20.5 × 5.0–6.0 µm (average = 17.0 × 5.3 µm, n = 45, L/W = 3.2), hyaline, thin-walled, smooth with granular contents, unicellular, aseptate narrowly fusiform, base subtruncate to bluntly rounded.

##### Culture characteristics.

Conidia germinate on PDA within 24 hours at room temperature (25 °C) with germ tubes produced from both ends of the conidia. Colonies with white fluffy mycelium on PDA (90 mm), after 7 days becomes raised, fluffy, white mycelium, dense filamentous.

**Figure 3.**
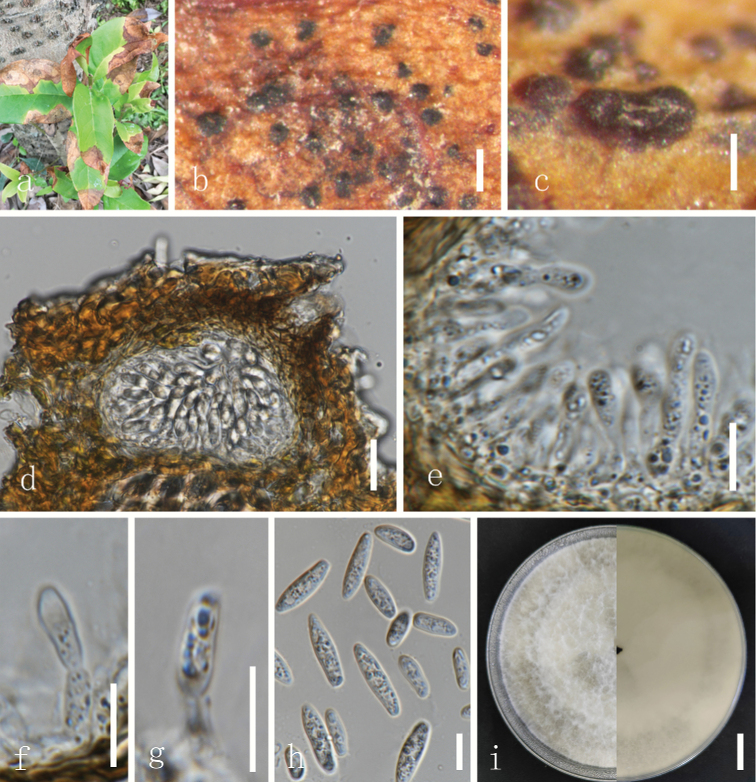
*Botryosphaeriaosmanthuse* (GUCC 21433, holotype) **a–c** colonies on natural substrate **d** section through conidiomata **e–g** conidiophores and conidia **h** conidia **i** colony on PDA (left: above, right: reverse). Scale bars: 300 µm (**b**); 140 µm (**c**); 50 µm (**d**); 20 µm (**e**); 10 µm (**f–h**); 15 mm (**i**).

##### Distribution.

China, Guangxi Province, Nanning City.

##### Other material examined.

China, Guangxi Province, Nanning City, 22°51'N, 108°19'E, from living leaves of *Osmanthusfragrans*, 20 October 2017, C.R. Meng, HGUP 21433, living culture GUCC 21433.1 and GUCC 21433.2.

##### Notes.

NCBI BLAST searches of ITS sequences from our strains suggest a high degree of similarity (99–100%) to *Botryosphaeriadothidea*. However, DNA bases in the two loci (*tef1* and *tub2*) showed a high amount of difference between *B.osmanthuse* and *B.dothidea*. *Botryosphaeriaosmanthuse* shows close phylogenetic affinity to *B.puerensis* (Fig. 1). Comparing the morphological characteristics, conidia of *B.osmanthuse* (av. 17.0 × 5.3; L/W = 3.2) are narrower and shorter than *B.puerensis* (av. 26.8 × 6.4; L/W = 4.2) (Li et al. 2020). *Botryosphaeriaosmanthuse* was first isolated from *Osmanthusfragrans* (Oleaceae), while *B.puerensis* has been reported from *Eucalyptusurophylla* (Myrtaceae).

## ﻿Discussion

Two new species of *Botryosphaeria*, *B.salicicola* and *B.osmanthuse*, are described and illustrated from southern China in this paper. Previously reported *Botryosphaeria* species in China are listed in Table 4. Thirteen *Botryosphaeria* species were described from nine different areas in southern China, covering three climatic zones (northern sub-tropical zone, central sub-tropical zone and warm temperate zone) along an altitudinal gradient (Hui 2021). Most species, such as *B.fabicerciana*, *B.fujianensis*, *B.fusispora*, *B.kuwatsukai*, *B.dolichospermatii*, *B.minutispermatia*, *B.pseudoramosa*, *B.qingyuanensis* and *B.wangensis*, often caused serious diseases on their hosts (Xu et al. 2015; Ariyawansa et al. 2016; Zhou et al. 2016, 2017; Li et al. 2018, 2020; Vu et al. 2019; Chen et al. 2020; Chu et al. 2021). Geographical and climatic regions have a large influence on the taxonomy, ecological distribution and pathogenicity of *Botryosphaeria* species (Phillips et al. 2013).

**Table 4.** List of Chinese *Botryosphaeria* strains.

Species	Strain	Host/ Natural substrate	Regions	Fungi	References
*Botryosphaeriafabicerciana*	CBS 127193	*Eucalyptus* sp.	Fujian	Pathogens	Li et al. (2018)
	CMW 27094	*Eucalyptus* sp.	Fujian	Pathogens	Li et al. (2018)
	CMW 27121	*Eucalyptus* sp.	Fujian	Pathogens	Li et al. (2018)
	CERC 2930	*Eucalyptus* sp.	Yunnan	Pathogens	Li et al. (2018)
	CERC 3446	*Eucalyptus* sp.	Guangdong	Pathogens	Li et al. (2018)
	CERC 2912	*E.urophylla* & *E.grandis*	Yunnan	Pathogens	Li et al. (2018)
	CERC 2913	*E.urophylla* & *E.grandis*	Yunnan	Pathogens	Li et al. (2018)
*B.fujianensis*	CGMCC 3.19099	*Vacciniumuliginosum*	Fujian	Pathogens	Chu et al. (2021)
	BJFUCC 180226-3	*V.uliginosum*	Fujian	Pathogens	Chu et al. (2021)
	BJFUCC 180226-4	*V.uliginosum*	Fujian	Pathogens	Chu et al. (2021)
*B.fusispora*	CSF6063	*E.urophylla* & *E.grandis*	Yunnan	Pathogens	Li et al. (2020)
	CSF6178	*E.globulus*	Yunnan	Pathogens	Li et al. (2020)
	CSF5872	*E.urophylla* & *E.grandis*	Yunnan	Pathogens	Li et al. (2020)
	CSF5950	*E.urophylla* & *E.grandis*	Yunnan	Pathogens	Li et al. (2020)
	CSF6160	*E.globulus*	Yunnan	Pathogens	Li et al. (2020)
	CSF6056	*E.urophylla* & *E.grandis*	Yunnan	Pathogens	Li et al. (2020)
*B.guttulata*	CGMCC3.20094	Decaying branch	Guizhou	Saprobes	Chen et al. (2020)
	GZCC 19-0186	Decaying branch	Guizhou	Saprobes	Chen et al. (2020)
	GZCC 19-0188	Decaying branch	Guizhou	Saprobes	Chen et al. (2020)
*B.kuwatsukai*	CBS 135219	*Malusdomestica*	Unknown	Pathogens	Xu et al. (2015)
	LSP 5	*Pyrus* sp.	Unknown	Pathogens	Xu et al. (2015)
*B.dolichospermatii*	CGMCC 3.19096	*V.uliginosum*	Fujian	Pathogens	Chu et al. (2021)
	CGMCC 3.19097	*V.uliginosum*	Fujian	Pathogens	Chu et al. (2021)
	GZCC 16-0013	Dead wood	Guizhou	Saprobes	Ariyawansa et al. (2016)
	GZCC 16-0014	Dead wood	Guizhou	Saprobes	Ariyawansa et al. (2016)
*B.pseudoramosa*	CERC 2001	*E.hybrid*	Guangxi	Pathogens	Li et al. (2018)
	CERC 2982	Unknow	Guangxi	Pathogens	Li et al. (2018)
	CERC 2983	*Melastomasanguineum*	Guangxi	Unsure	Li et al. (2018)
	CGMCC 3.18739	*Eucalyptus* sp.	Guangxi	Unsure	Li et al. (2018)
	CERC 3462	*Eucalyptus* sp.	Guangxi	Unsure	Li et al. (2018)
	CERC 2019	*E.urophylla* & *E.grandis*	Guangxi	Unsure	Li et al. (2018)
	CERC 2987	*Me.sanguineum*	Guangxi	Unsure	Li et al. (2018)
	CERC 3455	*Eucalyptus* sp.	Guangxi	Unsure	Li et al. (2018)
	CERC 2988	*Me.sanguineum*	Guangxi	Unsure	Li et al. (2018)
*B.qingyuanensis*	CERC 2946	*E.hybrid*	Guangdong	Pathogens	Li et al. (2018)
	CERC 2947	*E.hybrid*	Guangdong	Pathogens	Li et al. (2018)
*B.ramosa*	CGMCC 3.18004	*Acacia* sp.	Hainan	Unsure	Vu et al. (2019)
	CGMCC 3.18006	*Myrtaceae*	Guangdong	Unsure	Vu et al. (2019)
*B.rosaceae*	CGMCC 3.18007	*Malus* sp.	Shandong	Unsure	Zhou et al. (2017)
	CGMCC 3.18008	*Amygdalus* sp.	Shandong	Unsure	Zhou et al. (2017)
	CGMCC3.18009	*Malus* sp.	Shandong	Unsure	Zhou et al. (2017)
	CGMCC3.18010	*Pyrus* sp.	Shandong	Unsure	Zhou et al. (2017)
	CFCC 82350	*Malus* sp.	Unknown	Unsure	Zhou et al. (2017)
	CGMCC3.18011	*Pyrus* sp.	Shandong	Unsure	Zhou et al. (2017)
*B.sinensia*	CGMCC 3.17722	*Populus* sp.	Henan	Unsure	Zhou et al. (2016)
	CGMCC 3.17723	*Morus* sp.	Henan	Unsure	Zhou et al. (2016)
	CGMCC 3.17724	*Juglansregia*	Henan	Unsure	Zhou et al. (2016)
	CFCC 82346	*J.regia*	Beijing	Unsure	Zhou et al. (2016)
	CFCC 82255	*Ma.pumila*	Beijing	Unsure	Zhou et al. (2016)
*B.wangensis*	CERC 2298	*C.deodara*	Henan	Pathogens	Li et al. (2018)
	CERC 2299	*C.deodara*	Henan	Pathogens	Li et al. (2018)
	CGMCC 3.18744	*C.deodara*	Henan	Pathogens	Li et al. (2018)
	CERC 2300	*C.deodara*	Henan	Pathogens	Li et al. (2018)
	CSF5820	*E.urophylla* & *E.grandis*	Yunnan	Pathogens	Li et al. (2020)
	CSF5733	*Eucalyptus* sp.	Yunnan	Pathogens	Li et al. (2020)
	CSF5944	*E.urophylla* & *E.grandis*	Yunnan	Pathogens	Li et al. (2020)
	CSF5971	*E.urophylla* & *E.grandis*	Yunnan	Pathogens	Li et al. (2020)
	CSF5781	*E.globulus*	Yunnan	Pathogens	Li et al. (2020)
	CSF6174	*E.globulus*	Yunnan	Pathogens	Li et al. (2020)
	CSF5737	*Eucalyptus* sp.	Yunnan	Pathogens	Li et al. (2020)
*B.archontophoenicis*	HKU (M) 3539	*Archontophoenixalexandrae*	Hong Kong	Saprobes	Index Fungorum and mycobank
*B.brunneispora*	HKU (M) 3987	*Trachycarpusfortune*	Hubei	Unsure	Index Fungorum and mycobank
*B.cunninghamiae*	N/A	*Cunninghamialanceolata*	China	Saprobes	Index Fungorum and mycobank
*B.puerensis*	HMAS 255719	*E.urophylla* & *E.grandis*	China	Pathogens	Index Fungorum and mycobank
*B.qinlingensis*	BJFC S1576	*Quercus aliena var. acuteserrata*	Shaanxi	Unsure	Index Fungorum and mycobank
*B.yedoensis*	N/A	*Prunusyedoensis*	Taiwan	Unsure	Index Fungorum and mycobank

*Botryosphaeria* species have been known to exist in many woody plants (Crous et al. 2006; Liu et al. 2012; Phillips et al. 2013; Ariyawansa et al. 2016; Dissanayake et al. 2016; Slippers et al. 2017). *Botryosphaeriadothidea*, the type species of the genus (Slippers and Wingfield 2007), is known from numerous hosts (Phillips et al. 2013; Marsberg et al. 2017) and was isolated from an *Asphondylia* gall on *Lamiaceae* in Italy and Poland (Zimowska et al. 2020). Other species of *Botryosphaeria* have often been isolated from many wood plants (Table 2). Amongst them, *B.fabicerciana*, *B.fusispora*, *B.kuwatsukai*, *B.pseudoramosa*, *B.rosaceae*, *B.wangensis* and *B.puerensis* often exist in many economic plants, such as *Eucalyptus* sp., *Pyrus* sp., *Malus* sp., *Citrus* sp. and *Vaccinium* sp. (Phillips et al. 2006; Lazzizera et al. 2008; Zhou et al. 2017; Li et al. 2018, 2020; Chen et al. 2020). Our strains were isolated from the *Salix* (Salicaceae) and *O.fragrans* (Oleaceae) of woody plants. In contrast, the few hosts or natural substrates of the known species belong to the Salicaceae and Oleaceae.

## Supplementary Material

XML Treatment for Botryosphaeria salicicola

XML Treatment for Botryosphaeria osmanthuse

## References

[B1] Ariyawansa HA Hyde KD Liu JK Wu SP Liu ZY (2016) Additions to Karst Fungi 1: *Botryosphaeriaminutispermatia* sp. nov., from Guizhou Province, China. Phytotaxa 275(1): 35–44. 10.11646/phytotaxa.275.1.4

[B2] Alves A Crous PW Correia A Phillips AJL (2008) Morphological and molecular data reveal cryptic species in *Lasiodiplodiatheobromae*. Fungal Diversity 28: 1–13.

[B3] Cesati V Notaris GD (1863) Schema diclassificazione degli sferiacei italici aschigeri piu’o meno appartenenti al genere Sphaerianell’antico significato attribuitoglide Persoon. Commentario della Società Crittogamologica Italiana 14: 177–240.

[B4] Chen SF Pavlic D Roux J Slippers B Xie YJ Wingfield MJ Zhou XD (2011) Characterization of Botryosphaeriaceae from plantation-grown *Eucalyptus* species in South China. Plant Pathology 60(4): 739–751. 10.1111/j.1365-3059.2011.02431.x

[B5] Chen YY Dissanayake AJ Liu ZY Liu JK (2020) Additions to Karst Fungi 4: *Botryosphaeria* spp. associated with woody hosts in Guizhou province, China including *B.guttulata* sp. nov. Phytotaxa 454(3): 186–202. 10.11646/phytotaxa.454.3.2

[B6] Chu RT Dou ZP He W Zhang Y (2021) Two novel species of *Botryosphaeria* causing stem canker of blueberries from China. Junwu Xuebao 40: 473–486. 10.13346/j.mycosystema.200333

[B7] Crous PW Slippers B Wingfield MJ Rheeder J Marasas WFO Phillips AJL Alves A Burgess T Barber P Groenewald JZ (2006) Phylogenetic lineages in the Botryosphaeriaceae. Studies in Mycology 55: 235–253. 10.3114/sim.55.1.235 18490983 PMC2104729

[B8] Dissanayake AJ Phillips AJL Li XH Hyde KD (2016) Botryosphaeriaceae: Current status of genera and species. Mycosphere: Journal of Fungal Biology 7(7): 1001–1073. 10.5943/mycosphere/si/1b/13

[B9] Glass NL Donaldson GC (1995) Development of primer sets designed for use with the PCR to amplify conserved genes from filamentous ascomycetes. Microbiology 61: 1323–1330. 10.1128/aem.61.4.1323-1330.1995 PMC167388 7747954

[B10] Hui QH (2021) Division of climatic zones during the peak of the Holocene Warm Period in China. Journal of Qinghai Normal University 3: 60–65. [Natural Science]

[B11] Katoh K Rozewicki J Yamada KD (2019) MAFFT online service: Multiple sequence alignment, interactive sequence choice and visualization. Briefings in Bioinformatics 20(4): 1160–1166. 10.1093/bib/bbx108 28968734 PMC6781576

[B12] Lazzizera C Frisullo S Alves A Phillips AJL (2008) Morphology, phylogeny and pathogenicity of *Botryosphaeria* and *Neofusicoccum* species associated with drupe rot of olives in southern Italy. Plant Pathology 57(5): 948–956. 10.1111/j.1365-3059.2008.01842.x

[B13] Li GQ Liu FF Li JQ Liu QL Chen SF (2018) Botryosphaeriaceae from *Eucalyptus* plantations and adjacent plants in China. Persoonia 40(1): 63–95. 10.3767/persoonia.2018.40.03 30504996 PMC6146638

[B14] Li GQ Slippers B Wingfield MJ Chen SF (2020) Variation in Botryosphaeriaceae from *Eucalyptus* plantations in Yunnan Province in southwestern China across a climatic gradient. IMA Fungus 11(1): 22. 10.1186/s43008-020-00043-x 33117629 PMC7560076

[B15] Liu YJ Whelen S Hall BD (1999) Phylogenetic relationships among ascomycetes: evidence from an RNA polymerase II subunit. Molecular Biology and Evolution 16: 1799–1808. 10.1093/oxfordjournals.molbev.a026092 10605121

[B16] Liu JK Phookamsak R Doilom M Wikee S Li YM Ariyawansha H Boonmee S Chomnunti P Dai DQ Bhat JD Romero AI Zhuang WY Monkai J Gareth Jones EB Chukeatirote E Ko TWK Zhao YC Wang Y Hyde KD (2012) Towards a natural classification of Botryosphaeriales. Fungal Diversity 57(1): 149–210. 10.1007/s13225-012-0207-4

[B17] Marsberg A Kemler M Jami F Nagel JH Smidt AP Naidoo S Wingfield MJ Crous PW Spatafora JW Hesse CN Robbertse B Slippers AB (2017) *Botryosphaeriadothidea*: A latent pathogen of global importance to woody plant health. Molecular Plant Pathology 18(4): 477–488. 10.1111/mpp.12495 27682468 PMC6638292

[B18] Nguyen LT Schmidt HA Von Haeseler A Minh BQ (2015) IQ-TREE: A fast and effective stochastic algorithm for estimating maximum-likelihood phylogenies. Molecular Biology and Evolution 32(1): 268–274. 10.1093/molbev/msu300 25371430 PMC4271533

[B19] Pennycook SR Samuels GJ (1985) *Botryosphaeria* and *Fusicoccum* species associated with ripe fruit rot of *Actinidiadeliciosa* (kiwifruit) in New Zealand. Mycotaxon 24: 445–458.

[B20] Phillips AJL Oudemans PV Correia A Alves A (2006) Characterisation and epitypification of *Botryosphaeriacorticis*, the cause of blueberry cane canker. Fungal Diversity 21: 141–155.

[B21] Phillips AJL Alves A Pennycook SR Johnston PR Ramaley A Akulov A Crous PW (2008) Resolving the phylogenetic and taxonomic status of dark-spored teleomorph genera in the Botryosphaeriaceae. Persoonia 21(1): 29–55. 10.3767/003158508X340742 20396576 PMC2846129

[B22] Phillips AJL Alves A Abdollahzadeh J Slippers B Wingfield MJ Groenewald JZ Crous PW (2013) The Botryosphaeriaceae: Genera and species known from culture. Studies in Mycology 76: 51–167. 10.3114/sim0021 24302790 PMC3825232

[B23] Phillips AJL Hyde KD Alves A Liu JK (2019) Families in Botryosphaeriales: A phylogenetic, morphological, and evolutionary perspective. Fungal Diversity 94(1): 1–22. 10.1007/s13225-018-0416-6

[B24] Ronquist F Teslenko M Mark VD Ayres P Darling DL Höhna A Larget S Liu B Suchard LMA Huelsenbeck JP (2012) MrBayes 3.2: Efficient Bayesian phylogenetic inference and model choice across a large model space. Systematic Biology 61(3): 539–542. 10.1093/sysbio/sys029 22357727 PMC3329765

[B25] Senanayake IC Rathnayake AR Marasinghe DS Calabon MS Gentekaki E Lee HB Hurdeal VG Pem D Dissanayake LS Wijesinghe SN Bundhun D Nguyen TT Goonasekara ID Abeywickrama PD Bhunjun CS Jayawardena RS Wanasinghe DN Jeewon R Bhat DJ Xiang MM (2020) Morphological approaches in studying fungi: Collection, examination, isolation, sporulation and preservation. Mycosphere: Journal of Fungal Biology 11(1): 2678–2754. 10.5943/mycosphere/11/1/20

[B26] Shoemaker RA (1964) Conidial states of some *Botryosphaeria* species on *Vitis* and *Quercus*. Canadian Journal of Botany 42(9): 1297–1301. 10.1139/b64-122

[B27] Slippers B Crous PW Denman S Coutinho T (2004) Combined Multiple Gene Genealogies and Phenotypic Characters Differentiate Several Species Previously Identified as *Botryosphaeriadothidea*. Mycologia 96: 83–101. 10.1080/15572536.2005.11833000 21148832

[B28] Slippers B Wingfield MJ (2007) Botryosphaeriaceae as endophytes and latent pathogens of woody plants: Diversity, ecology and impact. Fungal Biology Reviews 21(2–3): 90–106. 10.1016/j.fbr.2007.06.002

[B29] Slippers B Crous PW Jami F Groenewald JZ Wingfield MJ (2017) Diversity in the Botryosphaeriales: Looking back, looking forward. Fungal Biology 121(4): 307–321. 10.1016/j.funbio.2017.02.002 28317537

[B30] Swofford DL (2002) PAUP*: Phylogenetic analysis using parsimony (and other methods), version 4.0 b10. MA: Sinauer Associates. Sunderland, UK.

[B31] Tamura K Stecher G Peterson D Filipski A Kumar S (2013) MEGA6: Molecular evolutionary genetics analysis version 6.0. Molecular Biology and Evolution 30(12): 2725–2729. 10.1093/molbev/mst197 24132122 PMC3840312

[B32] Trifinopoulos J Nguyen LT von Haeseler A Minh BQ (2016) W-IQ-TREE: A fast online phylogenetic tool for maximum likelihood analysis. Nucleic Acids Research 44(W1): W232–W235. 10.1093/nar/gkw256 PMC4987875 27084950

[B33] Vaidya G Lohman DJ Meier R (2011) SequenceMatrix: Concatenation software for the fast assembly of multi-gene datasets with character set and codon information. Cladistics 27(2): 171–180. 10.1111/j.1096-0031.2010.00329.x 34875773

[B34] Vu D Groenewald M Vries MD Stielow B Eberhardt U Al-Hatmi A Groenewald JZ Cardinali G Houbraken J Boekhout T Crous PW Robert V Verkley GJM (2019) Large-scale generation and analysis of filamentous fungal DNA barcodes boosts coverage for kingdom fungi and reveals thresholds for fungal species and higher taxon delimitation. Studies in Mycology 92(1): 135–154. 10.1016/j.simyco.2018.05.001 29955203 PMC6020082

[B35] White TJ Bruns T Lee S Taylor JW (1990) Amplification and direct sequencing of fungal ribosomal RNA genes for phylogenetics. In: Innes MA Gelfand DH Sninsky JJ White TJ (Eds) PCR protocols: a guide to methods and applications.Academic Press, San Diego, CA, 315–322. 10.1016/B978-0-12-372180-8.50042-1

[B36] Xu C Wang C Ju LL Zhang R Biggs AR Tanaka E Li B Sun GY (2015) Multiple locus genealogies and phenotypic characters reappraise the causal agents of apple ring rot in China. Fungal Diversity 71(1): 215–231. 10.1007/s13225-014-0306-5

[B37] Yukako H Yuho A Atsuko S Nami U Chiharu N (2021) Taxonomical study of noteworthy species of *Botryosphaeria* in Japan. Mycobiology 49(2): 122–132. 10.1080/12298093.2021.1895486 PMC10635109 37970183

[B38] Zhou YP Dou ZP He W Zhang Y Zhang Y (2016) *Botryosphaeriasinensia* sp. nov., a new species from China. Phytotaxa 245(1): 43–50. 10.11646/phytotaxa.245.1.4

[B39] Zhou YP Zhang M Dou ZhP Zhang Y (2017) *Botryosphaeriarosaceae* sp. nov. and *B.ramosa*, new botryosphaeriaceous taxa from China. Mycosphere: Journal of Fungal Biology 8(2): 162–171. 10.5943/mycosphere/8/2/2

[B40] Zimowska B Okoń S Becchimanzi A Krol ED Nicoletti R (2020) Phylogenetic characterization of *Botryosphaeria* strains associated with *Asphondylia* galls on species of Lamiaceae. Diversity (Basel) 12(2): 41. 10.3390/d12020041

